# A comparative study between deep learning and radiomics models in grading liver tumors using hepatobiliary phase contrast-enhanced MR images

**DOI:** 10.1186/s12880-022-00946-8

**Published:** 2022-12-14

**Authors:** Lixin Du, Jianpeng Yuan, Meng Gan, Zhigang Li, Pan Wang, Zujun Hou, Cong Wang

**Affiliations:** 1 Medical Imaging Department, Shenzhen Longhua District Central Hospital, Shenzhen, China; 2 Department of Radiology, The Seventh Affiliated Hospital, Sun Yat-sen University, Shenzhen, China; 3 Jiangsu Key Laboratory of Medical Optics, Suzhou Institute of Biomedical Engineering and Technology, Chinese Academy of Sciences, Suzhou, China; 4 Jinan Guoke Medical Technology Development Co., Ltd, Jinan, China

**Keywords:** Hepatocellular carcinoma, Radiomics, Deep learning, Magnetic resonance imaging

## Abstract

**Purpose:**

To compare a deep learning model with a radiomics model in differentiating high-grade (LR-3, LR-4, LR-5) liver imaging reporting and data system (LI-RADS) liver tumors from low-grade (LR-1, LR-2) LI-RADS tumors based on the contrast-enhanced magnetic resonance images.

**Methods:**

Magnetic resonance imaging scans of 361 suspected hepatocellular carcinoma patients were retrospectively reviewed. Lesion volume segmentation was manually performed by two radiologists, resulting in 426 lesions from the training set and 83 lesions from the test set. The radiomics model was constructed using a support vector machine (SVM) with pre-defined features, which was first selected using Chi-square test, followed by refining using binary least absolute shrinkage and selection operator (LASSO) regression. The deep learning model was established based on the DenseNet. Performance of the models was quantified by area under the receiver-operating characteristic curve (AUC), accuracy, sensitivity, specificity and F1-score.

**Results:**

A set of 8 most informative features was selected from 1049 features to train the SVM classifier. The AUCs of the radiomics model were 0.857 (95% confidence interval [CI] 0.816–0.888) for the training set and 0.879 (95% CI 0.779–0.935) for the test set. The deep learning method achieved AUCs of 0.838 (95% CI 0.799–0.871) for the training set and 0.717 (95% CI 0.601–0.814) for the test set. The performance difference between these two models was assessed by *t*-test, which showed the results in both training and test sets were statistically significant.

**Conclusion:**

The deep learning based model can be trained end-to-end with little extra domain knowledge, while the radiomics model requires complex feature selection. However, this process makes the radiomics model achieve better performance in this study with smaller computational cost and more potential on model interpretability.

## Introduction

Hepatocellular carcinoma (HCC) is a common type of cancer and a leading cause of cancer death worldwide [1]. The prognosis of HCC depends largely on the stage of the tumor. Multiphasic computed tomography (CT) and magnetic resonance imaging (MRI) have been endorsed as first-line modalities for non-invasive diagnosis and staging of liver tumors [2]. A number of studies have been conducted on the relationship between imaging features with HCC histopathologic grading [3–6], and the liver imaging reporting and data system (LI-RADS) is a widely-used technique to provide standardized criteria for performing, interpreting, and reporting multiphase CT and MRI exams for HCC diagnosis [7].

Recently, with the flourishing of radiomics techniques in tumor analysis [8–12], there is increasing interest in the development of imaging-based system for automated liver tumor grading [13–17], where machine learning methods were employed to find potential pattern from images in differentiation of liver tumor grade. A popular technique as employed in these studies to build the computer-aided systems is the method of deep learning [18, 19], which adds more layers into conventional neural network architecture and represents data in a hierarchical form to capture the complexity in the data. In spite of good performance as reported, deep learning-based systems in medical imaging application have been of debate in terms of size of dataset, computational cost and model interpretability [20–22].

In general, deep learning is a subdomain of machine learning, which includes supervised learning and unsupervised learning, and usually consists of two key components: features and classifiers. Common features include image spatial domain feature such as texture information, tumor shape characteristics, image intensity statistics, or image feature in a transform domain such as wavelet feature. Primary classifiers include decision tree, naive Bayes, neural network, support vector machine (SVM), etc. The basic classifiers can be ensembled to arrive at a classifier with improved performance, as represented by random forest and AdaBoost. Different machine learning methods have different advantages and disadvantages. Interested readers can refer to [23, 24] for a recent review on the application of machine learning to medical imaging.

In cancer imaging, it is unlikely to have a dataset of scale in applications such as face recognition or internet-based image recognition, hence, it is of interest to build systems taking into account the intrinsic property of medical imaging data and choosing machine learning algorithm with less requirement on the amount of data. This study attempted to compare a conventional radiomics model based on SVM with respect to a deep learning based model using DenseNet [25] for liver tumor grading.

## Materials and methods

**Fig. 1:**
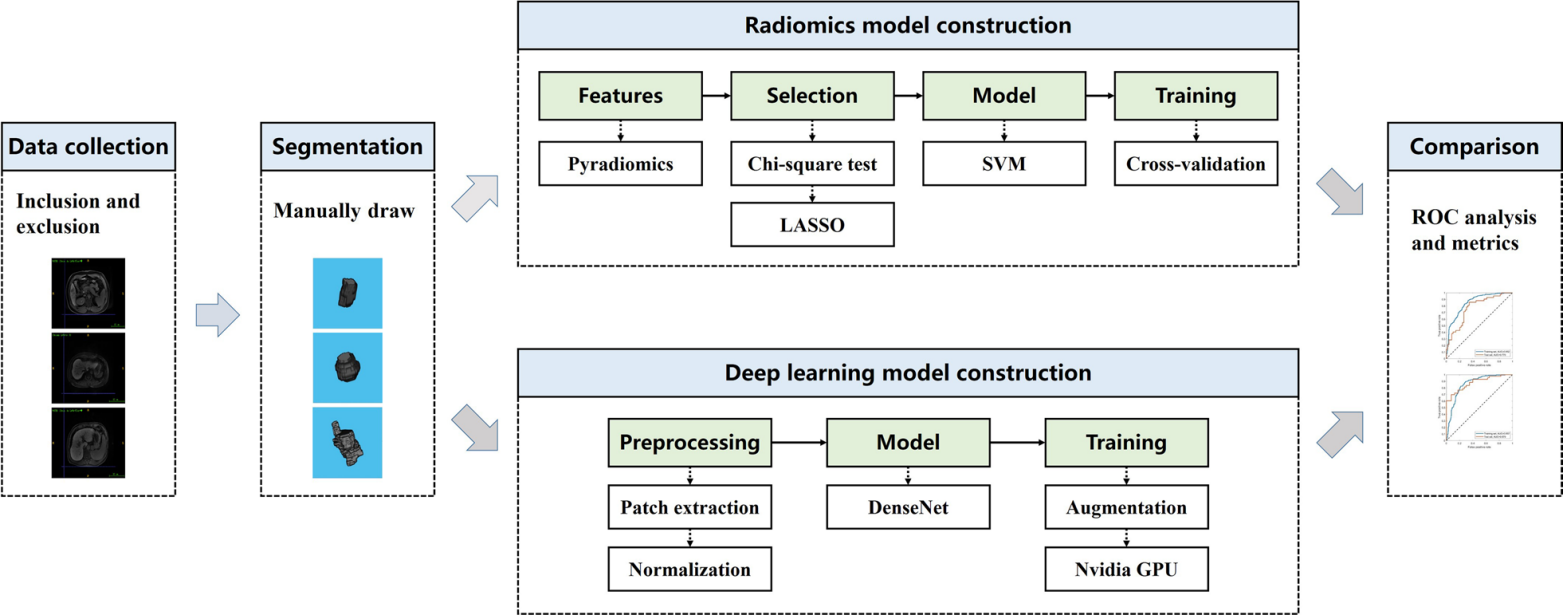
The workflow of this study

The workflow of this study was illustrated in Fig. 1, as detailed in the following.

### Study participants

This retrospective study was approved by the Ethical Committee of Shenzhen Longhua District Central Hospital. A total of 462 patients with suspected HCC who underwent examination between June 2016 and June 2021 were reviewed and included in this study. Inclusion criteria were: (1) patients with clinical reports; (2) patients with contrast enhanced MRI (CE-MRI) examination; (3) staging of liver tumors confirmed by radiologists; (4) no history of other types of tumor. Exclusion criteria were: (1) incomplete clinical results; (2) incomplete CE-MRI scan or unsatisfactory image quality due to patient movement.

### Data acquisition

All scans were performed using a 3.0T MRI scanner (Skyra, Siemens, Germany) with a sixteen-channel phase array coil that covered the entire liver. Routine MRI protocols included a respiratory-triggered fat-suppressed T1-weighted dual-echo sequence (repetition time [TR] 4.5 ms, echo time [TE] 1.29 ms and 2.52 ms, slice thickness 3 mm, gap of slice 0.6 mm, field of view [FOV] 380 mm $\times$ 340 mm, matrix 195 $\times$320), a respiratory-triggered fat-suppressed T2-weighted fast spin-echo sequence (TR 3000 ms, TE 84 ms, slice thickness 5 mm, gap of slice 1 mm, FOV 380 mm $\times$ 380 mm, matrix 320 $\times$ 320) and a diffusion-weighted sequence (b values 0, 400, 1000 s/$\text {mm}^2$, TR 3500 ms, TE 59 ms, slice thickness 5 mm, gap of slice 1mm, FOV 380 mm $\times$ 340 mm, matrix 108 $\times$ 128). Contrast agents (Gadolinium-ethoxybenzyl-diethylenetriamine pentaacetic acid [Gd-EOB-DTPA]; Primovist, Bayer) were administered at rate of 2 mL/sec and 0.1 mmol gadolinium per kilogram followed by 20 ml saline infusion. The early hepatic arterial phase was omitted. The post-contrast scan was performed at 30 s (arterial phase), 60 s (portal venous phase), 180 s (transitional phase) and 20 mins (hepatobiliary phase) after the injection of contrast agent using T1-weighted 3D gradient echo sequence with fat saturation and volumetric interpolated breath-hold examination and the parameters were: slice thickness 6 mm, TR 3.12 ms, TE 1.51 ms, matrix 290$\times$ 290, flip angle $10^\circ$.

### Image segmentation and LI-RADS grading

The images in hepatobiliary phase were employed for analysis in this study. Segmentation of images into volume of interest (VOI) that covered lesions was performed manually by two radiologists (with 6 and 10 years of experience) using ITK-SNAP (version 3.8.0), while blinded to histopathological results. Each case was first drawn by one radiologist and then reviewed by the other one to ensure high-quality final segmentation results.

**Fig. 2:**
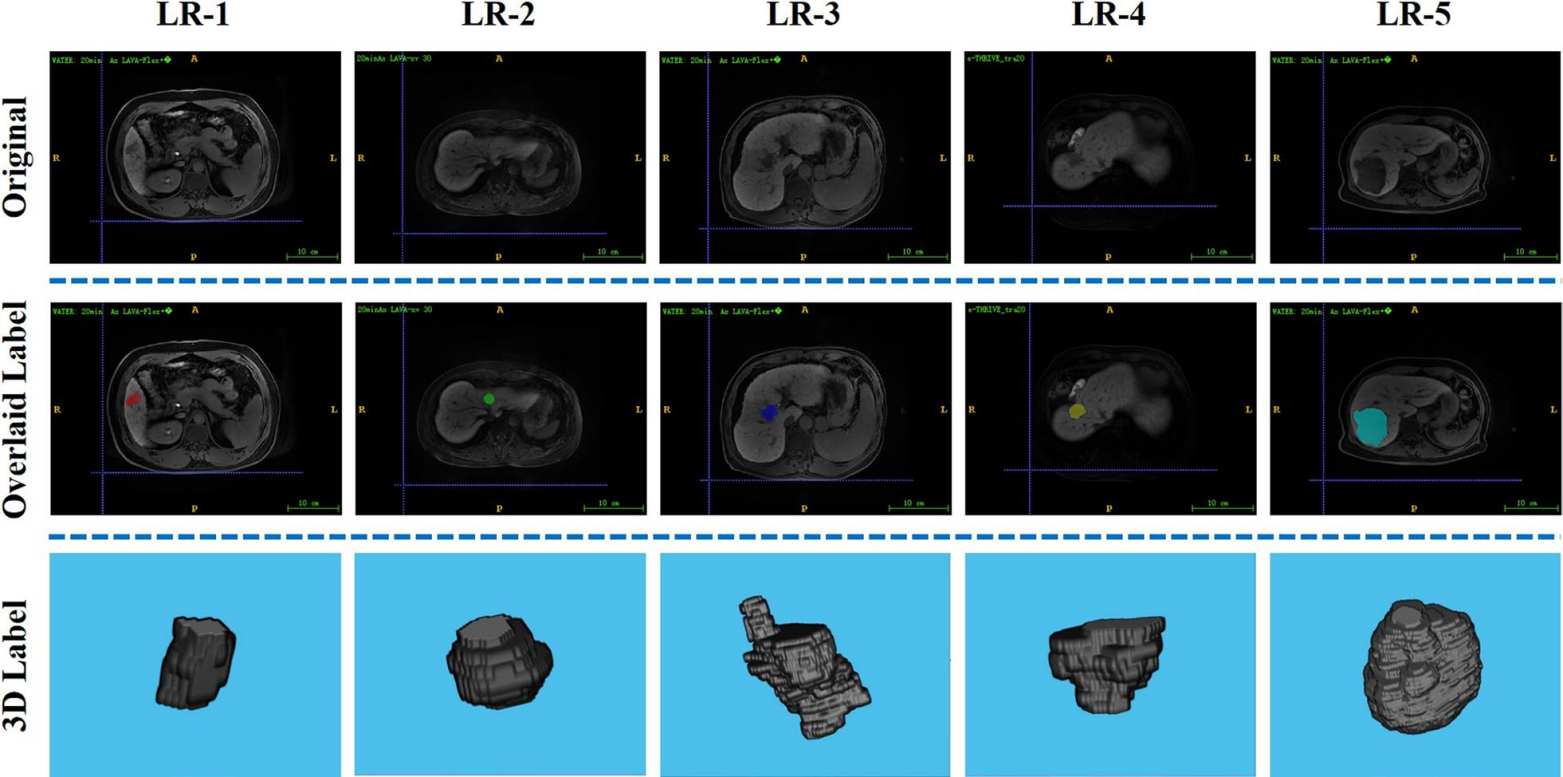
Samples of images from the selected patients with different LI-RADS categories

Then, these two radiologists independently analyzed all magnetic resonance (MR) images for assessing major and ancillary features, and assigned an LI-RADS category for each lesion according to the LI-RADS v2018 criteria [7]. Disagreements regarding the LI-RADS categorization were resolved by consensus with a senior abdominal radiologist with over 25 years of liver imaging experience. According to the LI-RADS diagnostic algorithm, a liver lesion is assigned a LI-RADS (LR) category (LR1 to LR5) to reflect the likelihood of being HCC. LR-1 and LR-2 are definitely benign and probably benign, respectively. LR-3 indicates intermediate probability of HCC, LR-4 indicates probable HCC, and LR-5 indicates definite HCC. In this study, the lesions are classified as low-grade tumor (LR-1 and LR-2) and high-grade tumor (LR-3, LR-4 and LR-5) for the following analysis. Samples of images from patients and the corresponding segmentation and grading results were shown in Fig. 2.

### Radiomics model construction

#### Radiomics feature extraction

Radiomics feature extraction based on the VOIs was performed using Pyradiomics software following the IBSI recommendation [26]. The VOIs were resampled with obtained isotropic voxels ($1 \times 1 \times 1$ mm). Normalization was performed by subtracting the mean value from each voxel and dividing by the standard deviation. A fixed bin width of 25 was used to compute textural features. Moreover, a Laplacian of Gaussian (LoG) filter (sigma values 1.5 mm and 2.5 mm) and wavelet decompositions (start level = 0, level = 1, wavelet = ”coif1”) were employed to accentuate textural differences in the VOIs. The extracted features included first-order features, shape-based features and higher-order texture features.

#### Feature selection

Feature reduction was performed using MATLAB (R2019b, Mathworks, Natick, USA). Chi-square test [27] was firstly used for univariate analysis, followed by binary least absolute shrinkage and selection operator (LASSO) regression [28] for multivariate analysis to achieve a proper combination of these radiomic features. *p*-value less than 0.1 was considered statistically significant in the Chi-square test.

#### Building of radiomics model

SVM [29] was employed to construct the model for distinguishing low-grade and high-grade liver tumors. In model training, 10-fold cross-validation was used for algorithm hyperparameter tuning based on the training set. During the process, we tested two kernels (linear and Gaussian) and four box constraint parameters *C* (1, 5, 10, 100). The parameter combination with the highest area under the receiver-operating characteristic curve (AUC) value was selected to build the final SVM model.

### Deep learning model construction

#### Data preprocessing

The VOIs were resampled to an isotropic resolution of voxel size ($1 \times 1 \times 1$ $\text {mm}^3$) and the intensity was rescaled to the range of 0 and 1. For each VOI, the volumes were zero-padded to the same size $100 \times 100 \times 100$ as the input for the deep networks, which covers the whole tumor region.

#### Model architectures

We employed the DenseNet for this task, which connects each layer to every other layer in a feed-forward fashion [25]. The DenseNet has several compelling advantages: it alleviates the vanishing-gradient problem, strengthens feature propagation, encourages feature reuse, and substantially reduces the number of parameters [25]. The network outputs a probability number ranging from 0 to 1 for each liver lesion, with a number closer to 1 indicating a higher probability that the lesion is high-grade liver tumor, whereas a number closer to 0 indicates a greater probability of being a low-grade liver tumor.

#### Training of the deep network

The DenseNet was implemented in Pytorch. Training of the network was performed on a 15 GB Nvidia A16 GPU using CUDA 11.1 with cuDNN v8.

The network was trained end-to-end using the Adam optimizer with Nesterov momentum of 0.9. An initial learning rate of $1 \times 10^{-4}$ was applied and was decayed by a factor of 10 if the validation loss failed to improve over ten consecutive epochs. Training was performed in batches of 16 randomly chosen samples at each iteration. After going through the entire training set, an epoch was finished, and 50 epochs were needed to accomplish the training. Finally, the model with the lowest validation loss was employed, which was used to measure the diagnostic performance of the network based on the test dataset.

During the training process, data augmentation was used to improve the network robustness. The data augmentation techniques used in this study included random rotation (range = 10 degrees), random shear (range = 0.05), random shift (range = 0.05) and random zoom (range = 0.05). Except for the random rotation range, the other parameters represent a fraction relative to the size of the corresponding dimension. When we feed images to the network during training, these augmentation techniques randomly operate on the input to provide images under different conditions, thereby improving network performance.

### Model evaluation

The effectiveness of the model was assessed on the independent test set. Receiver operating characteristic (ROC) curve and metrics including precision, recall and F1-score were used to assess the diagnostic performance [30, 31]. Difference between models was evaluated using *t*-test and *p* value less than 0.05 was considered statistically significant.

ROC curve for DenseNet can be calculated based on the value generated by the final sigmoid activate function, which can be regarded as a probabilistic output. As for the SVM, the output can be expressed as Eq. (1),

1 $$\begin{aligned} y = \text {sgn} (f({{\textbf {x}}})) = \text {sgn} (h({{\textbf {x}}}) + b) \end{aligned}$$

where sgn indicates the sign function. $h({{\textbf {x}}})$ is defined in Eq. (2), where *K* is the kernel function [32].

2 $$\begin{aligned} h({{\textbf {x}}}) = \sum _i y_i \alpha _i K({{\textbf {x}}}_i, {{\textbf {x}}}) \end{aligned}$$

In this study, we mapped the unthresholded outputs $f({{\textbf {x}}})$ into probabilities using an additional sigmoid function with the strategy proposed by Platt [32]. With the output probabilities, the ROC curve for SVM can be achieved.

**Table 1:** Characteristics of included patients

Item	Training	Test
Patient	293	68
Lesion	426	83
Low-grade	236	40
LR-1	111	19
LR-2	125	21
High-grade	190	43
LR-3	22	10
LR-4	35	7
LR-5	133	26

**Table 2:** Metrics for the proposed model on different data sets

Model	Data set	Accuracy	Precision	Recall	F1-score	AUC (95% CI)
Radiomics	Training	0.798	0.757	0.805	0.781	0.857 (0.816–0.888)
	Test	0.759	0.717	0.884	0.792	0.879 (0.779–0.935)
Deep	Training	0.770	0.775	0.815	0.794	0.838 (0.799–0.871)
	Test	0.659	0.617	0.690	0.652	0.717 (0.601–0.814)

*AUC* area under the curve, *CI* confidence interval

## Results

### Study participants

Among the 462 patients reviewed, 101 patients were excluded and 361 patients formed the study cohort (267 males and 94 females, age with mean 48.6 years and range 22 to 77 years). These HCC patients were divided into the training and the test set, which included 293 and 68 patients, respectively. From the training set, 426 lesions were segmented, and 83 lesions were from the test set. These lesions were graded based on LI-RADS v2018 and detailed patient demographics were listed in Table 1.

### Feature extraction and selection for the radiomics model

**Fig. 3:**
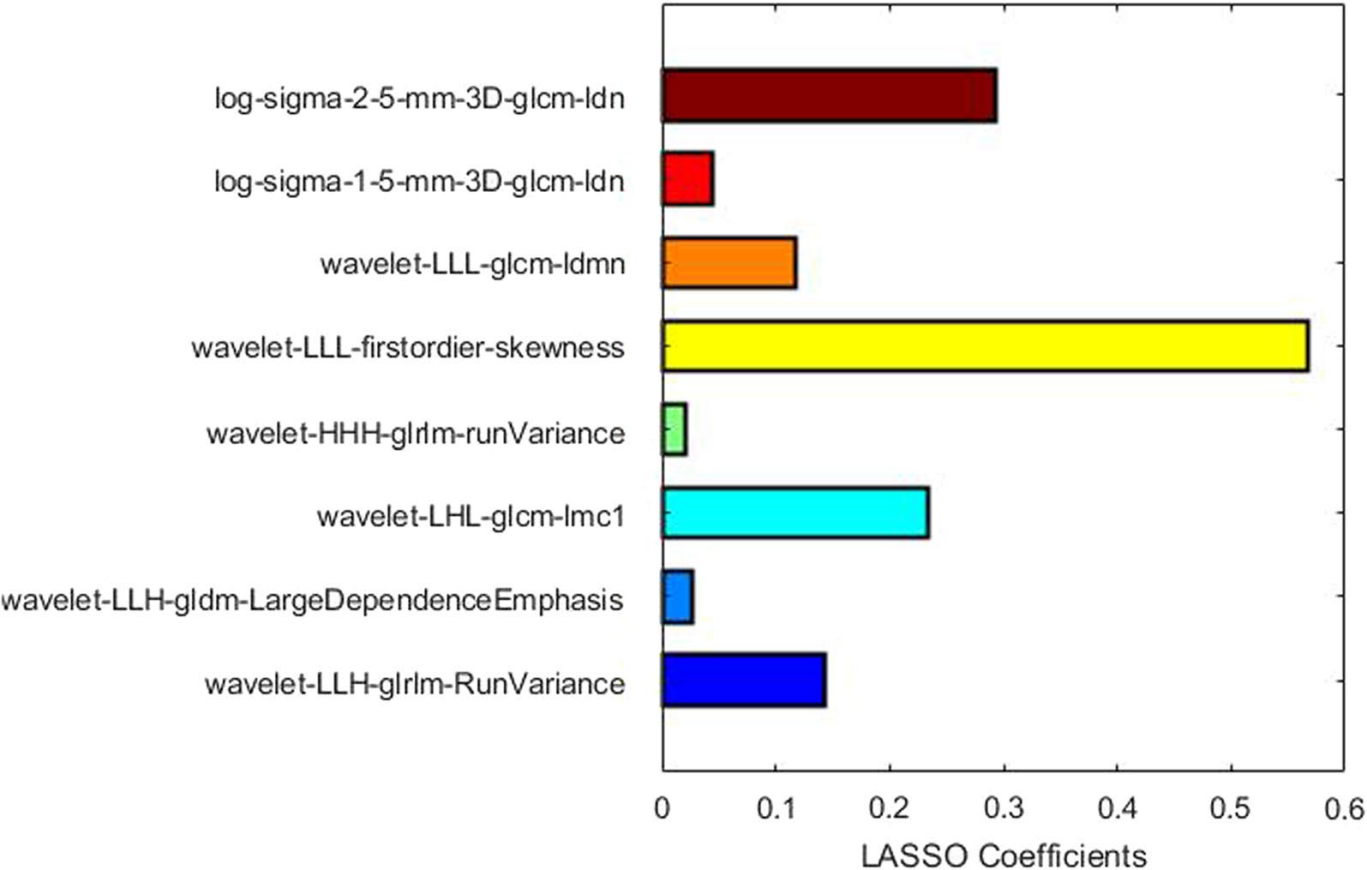
Features selected by the LASSO regression

A total of 1049 features were extracted for each VOI. After univariate analysis, 645 features were regarded as statistically significant ($p < 0.1$). The LASSO regression demonstrated that 8 features were effective indicators for discriminating low-grade and high-grade lesions, as illustrated in Fig. 3.

### Performance and comparisons of the two models

**Fig. 4:**
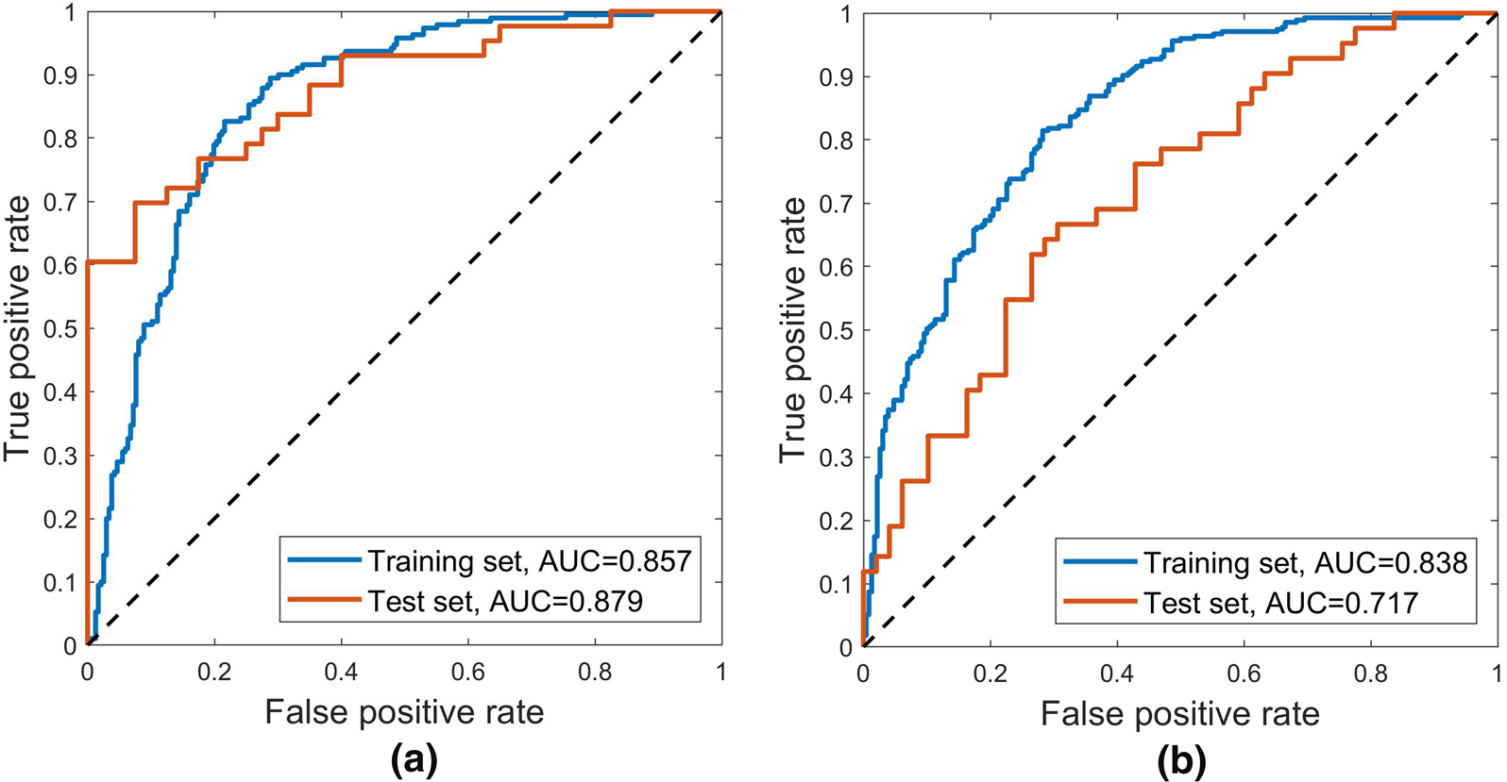
ROC curves of **a** the radiomics model and **b** the deep learning model

In this study, the SVM model uses the linear kernel with box constraint parameter *C* set to 5. The ROC curves of the radiomics model for the training and the test set were shown in Fig. 4a. The AUCs in differentiating low-grade from high-grade liver tumors were 0.857 (95% confidence interval [CI]: 0.816–0.888) for the training set, 0.879 (95% CI 0.779–0.935) for the test set. As for the deep learning model, the ROC curves were shown in Fig. 4b. The AUCs were 0.838 (95% CI 0.799–0.871) for the training set and 0.717 (95% CI 0.601–0.814) for the test set. More detailed metrics to quantify the model performance were listed in Table 2. The two models attained similar performance in differential diagnosis for the training set where the radiomics model only achieves slightly higher AUC than the deep learning model. As for the test set, the radiomics model shows much better performance, indicating the robustness of the system. The *t*-test shows that the results in both the training and the test set were statistically significant (p < 0.001), which further confirmed a better performance of the radiomics model compared to the deep learning model.

## Discussion

This study compared a deep learning model with a radiomics model in differentiating high-grade liver tumors from low-grade ones. The result showed that the two models attained close performance in the training set with similar metric values (Table 2) and ROC curves (Fig. 4), which indicated that both methods could find effective manifolds for describing the training data space. However, the radiomics model performs much better in the test set. One reason could be that the deep network was trained based on the whole volumes of size $100 \times 100 \times 100$, which includes intensities of 1,000,000 voxels regarded as features. Although 426 lesions were employed for training, the data size was still small for the large input features and the complex architecture, which limited the generalization ability of the model. On the contrary, the radiomics model was established using only 8 selected radiomics features, and the intrinsic manifold was much easier to model than the original volumes, thus resulting in a more robust predicting system.

In this study, the SVM is used to build the radiomics model. The SVM is effective in high dimensional space and was widely used in the field of medical image diagnosis [9, 10]. Besides, it uses a subset of training points in the decision function, which makes it memory efficient. The deep learning model is established using DenseNet, which won the best paper award in CVPR2017 [25]. DenseNet has several compelling advantages: they alleviate the vanishing-gradient problem, strengthen feature propagation, encourage feature reuse, and substantially reduce the number of parameters [25]. As a result, these two representative methods were employed in this study to provide convincing comparisons.

The requirement of sample size in deep learning was studied in [33] and it was pointed out that for single hidden-layer feedforward neural networks with number of units *k*, number of weights *d* and classification error $\epsilon$ ($0 < \epsilon \le 1/8$), the required number of training images *m* could be estimated as $m \le O(d/\epsilon , \log _2(k/\epsilon ))$. In practice, the architecture of deep networks involves many hidden layers, leading to large number of parameters needed to be estimated, which in turn increases the requirement on the size of training data. The method of deep learning model in this study was implemented using data augmentation to alleviate this issue, attaining AUC of 0.838 (0.799–0.871) and 0.717 (0.601–0.814) in liver tumor grading for the training and the test set. Zhou et al. [34] proposed a deep neural network by combining the squeeze-and-excitation networks in a three-dimensional densely connected convolutional network, and validated the method in a dataset consisting of 213 HCC patients of contrast-enhanced MR images, attaining AUC of 0.83, which is close to the result of the deep network implemented in this study. Nevertheless, If the training samples are from a data distribution that is very different from the one met in the real world, then the network’s generalization performance will be lower than expected [35].

Another major problem associated with deep learning is the issue of black box, as the complicated hierarchical representation of training data makes the prediction very difficult to understand and interpret [36]. How to enhance the interpretability of machine learning system has become a young but rapidly growing body of research in the domain of artificial intelligence (AI), and a lot of research efforts have been dedicated to this direction, including the explainable AI program launched by the defense advanced research projects agency (DARPA). Majority of these efforts focus on enhancing the transparency of machine learning systems through visualizing features learned in the training process and providing insight into the working of deep neural networks. Interested readers can refer to [37] for a recent survey on interpretable machine learning. Nevertheless, the research on interpretable deep learning remains in an early stage. Neurons might learn features without equivalent human concepts, and visualized features do not mean that the underlying reasoning mechanism of deep neural nets would be disclosed explicitly.

By comparison, conventional machine learning methods could be much easier to understand. For example, a decision tree establishes the classification based on a set of “if/else” rules, which is very interpretable. An SVM model represents different classes in a hyperplane in multidimensional space and divides the datasets into classes through maximizing the margin of hyperplane. Methods have been developed to facilitate the interpretability of SVMs by visualizing results as nomograms, such as the nomogram method using decomposable kernels in SVMs [38], the nomogram representation by replacing the lines by color bars with colors offering the same interpretation as the length of the lines in conventional nomograms [39]. In addition, non-image features such as clinical findings could easily be integrated into conventional machine learning systems, which is particularly important in the development of medical AI solution. SVM is very flexible in handling multiple continuous and categorical variables.

One more advantage of conventional machine learning methods is the cost of computation, which is generally more cost-saving in conventional models than in deep-learning methods. In this study, training the SVM classifier took less than 5 s in MATLAB with a personal computer (Intel Core i7-6700 CPU, 16 GB RAM). In comparison, achieving a well trained DenseNet took about 1 h using the 15 GB Nvidia GPU. The high efficiency of SVM-based radiomics model could be attributed to the use of hand-crafted features, whereas the end-to-end deep learning strategy saved human intervention at the cost of computational resources.

The VOI that covered lesions was obtained by manual segmentation. The procedure is performed by two radiologists. Each case was first drawn by one radiologist and then reviewed by the other one to reduce subjectivity. An alternative approach is using the STAPLE [40] tool that can produce a consolidated reference between different operators. Although two radiologists were invited to ensure a high-quality result, limitations of manual delineation still exist. As reported in [41], manual segmentation is labor-intensive, time-consuming, and not always feasible for radiomics analysis requiring huge datasets. Additionally, manual segmentation is subject to inter- and intra-observer variability [42]. One solution is using semi-automatic delineation algorithms, such as region growing or thresholding. These methods may be less precise than manual segmentation, but are more efficient with better reproducibility.

This study was limited in the design of experiment which included only the contrast-enhanced MR images, and did not incorporate other information such as other imaging modalities, or clinical findings. As the present study aimed to compare the efficacy between deep learning models and conventional machine learning methods, the focus of experiment design was to ensure a fair comparison rather than to arrive at an optimal liver tumor grading solution. As discussed in [43], HCC examinations should include late hepatic arterial, portal venous, and, at about 3–5 min, delayed phase acquisitions. In addition, liver tissue microenvironment could be quantitatively derived using dynamic contrast-enhanced imaging [44], and it would be straightforward to integrate such information in the framework of the SVM-based radiomics model.

In conclusion, this study compared a deep learning model with respect to the radiomics model in differentiating high-grade liver tumors from low-grade tumors, and showed that simple machine learning method could attain better performance than the deep learning method in liver tumor grading, with much smaller computational cost. As machine learning problems in medical imaging are featured by limited number of patient data and high requirement on interpretability of intelligent solutions, it would be recommended to explore the issue with conventional and more understandable machine learning methods.

## Data Availability

The datasets used or analyzed during the current study available from the corresponding author on reasonable request.
